# Case report: Motor neuron disease phenotype associated with symptomatic copper deficiency: Challenging diagnosis and treatment

**DOI:** 10.3389/fneur.2022.1063803

**Published:** 2023-01-04

**Authors:** Adam Benkirane, Thibault Warlop, Adrian Ivanoiu, Pierre Baret, Elsa Wiame, Vincent Haufroid, Thierry Duprez, Philippe Hantson

**Affiliations:** ^1^Department of Neurology, Cliniques Universitaires St-Luc, Brussels, Belgium; ^2^Institute of Neurosciences, Université Catholique de Louvain, Brussels, Belgium; ^3^Hospital Pharmacy, Cliniques Universitaires St-Luc, Brussels, Belgium; ^4^Laboratory of Physiological Chemistry, Université Catholique de Louvain and the Christian de Duve Institute of Cellular Pathology, Brussels, Belgium; ^5^Laboratory of Toxicology, Cliniques Universitaires St-Luc, Brussels, Belgium; ^6^Louvain Centre for Toxicology and Applied Pharmacology, Université Catholique de Louvain, Brussels, Belgium; ^7^Department of Neuroradiology, Cliniques Universitaires St-Luc, Brussels, Belgium; ^8^Department of Intensive Care, Cliniques Universitaires St-Luc, Brussels, Belgium

**Keywords:** copper deficiency, myelopathy, cerulopasmin, motoneuron, amyotrophic lateral sclerosis (ALS)

## Abstract

Copper deficiency is an acquired condition that can lead to neurologic dysfunctions, such as myelopathy, motor neuron impairment, polyneuropathy, cognitive impairment, and optic nerve neuropathy. Associated biological findings are low serum copper and ceruloplasmin levels with low copper urinary excretion. We report the case of a previously healthy 59-year-old man who presented a complex neurological picture starting with symptoms and radiological signs consistent with degenerative myelopathy in the presence of persisting low serum copper and ceruloplasmin despite oral and intravenous copper supplementation. Over time, his symptoms evolved into a motor neuron disease evocating an amyotrophic lateral sclerosis (ALS) phenotype. The potential role of copper deficiency is discussed, together with the difficulties in biomonitoring copper supplementation.

## Introduction

Copper is a mineral micronutrient playing a key role in numerous cellular processes. Copper is found in a wide range of foods, from meat to vegetables. In adults, average daily intakes of copper from food are 1,400 mcg for men and 1,100 mcg for women (1). Copper is primarily absorbed in the stomach, the duodenum, and the jejunum. The main causes of acquired copper deficiency are alteration of bowel absorption related to bariatric surgery, malabsorption syndromes, prolonged parenteral alimentation, and copper-chelating medications (2–4). Excessive zinc ingestion is also linked with this condition, with zinc and copper being competitively absorbed in the gastrointestinal tract (5). Chronic hemodialysis or dental prosthesis paste ingestion can also cause zinc overload (5, 6). Clinical manifestations of copper deficiency are primarily hematological and neurological (7). Copper deficiency-related myelopathy is a documented but often underdiagnosed entity characterized by the subacute onset of latero-dorsal spinal column dysfunction (8–11). The time from onset to diagnosis can vary from months to years. Ataxia is the most frequent neurological manifestation, while other symptoms are mild leg spasticity, neuropathy, cognitive impairment, and optic nerve neuropathy (8). The diagnosis of copper deficiency relies on the laboratory determination of copper and ceruloplasmin. As illustrated by the herein reported case, and despite extensive investigations, the etiology of copper deficiency remains unknown in a significant percentage of cases. In documented cases of copper deficiency-related neurological disorders, the outcome is inconstantly influenced by copper supplementation.

## Case observation

**Figure 1:**
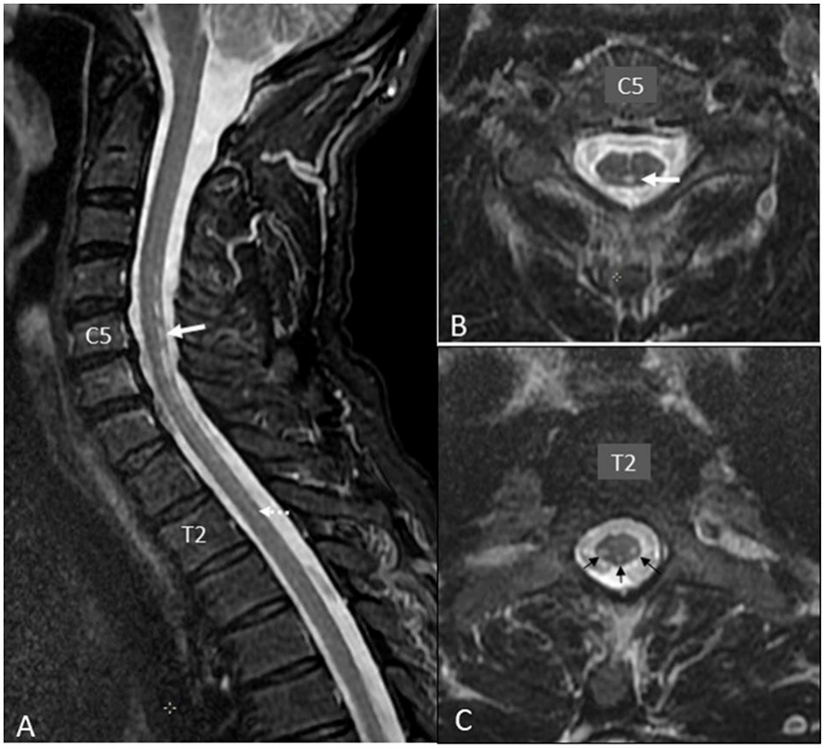
Spinal MR imaging (MRI) workup. **(A)** Mid-sagittal T2-weighted view from C0 to T5 showing linear hypersignal intensity within posterior columns prominently at the level of C5–C6 (white arrow). At the thoracic level, only faint signal intensity changes were suspected (dotted arrow at T2 level). **(B)** Axial transverse T2-weighted view at C5 level showing the posterior mid-sagittal focus of abnormal hypersignal intensity (arrow) reflecting elective tissue damage to the posterior columns. **(C)** Axial transverse T2-weighted view at T2 level showing decrease in size of tissue damage within posterior columns (middle arrow), but the appearance of abnormal hypersignal intensity within both crossed cortico-spinal tracts (lateral arrows), which were mostly outside slice location on mid-sagittal T2 view (1A).

**Figure 2:**
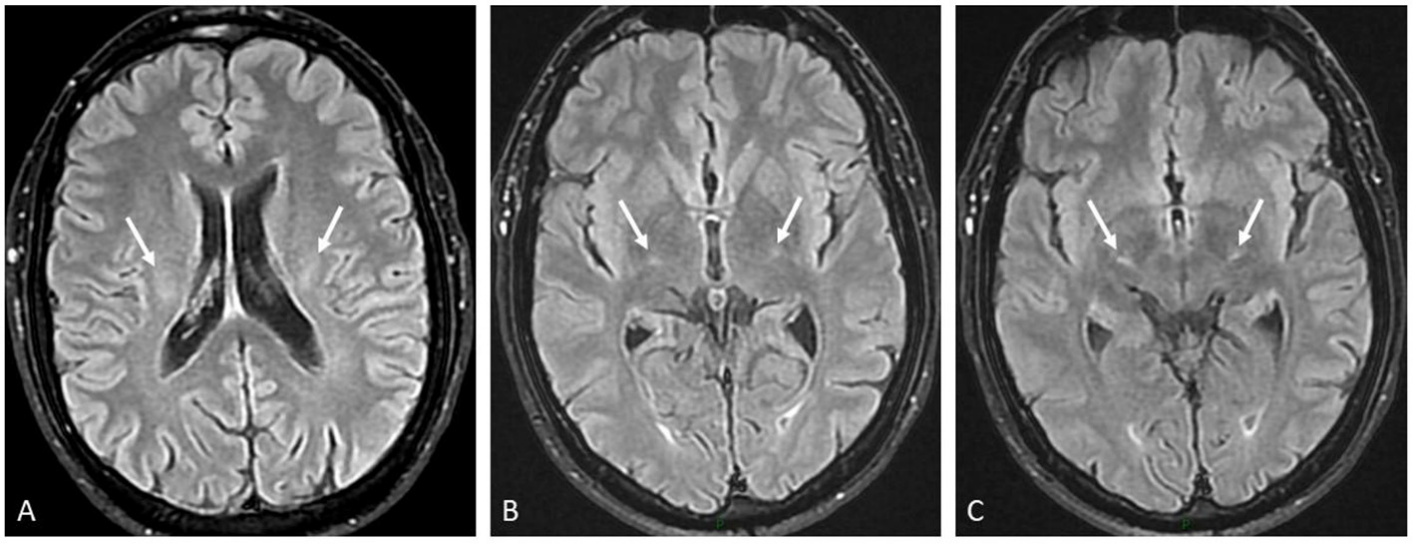
Brain MRI workup. Three axial transverse FLAIR views from the top to bottom showing moderate and symmetrical hypersignal intensity of the corticospinal tract (arrows) at the levels of the following: **(A)** the corona radiata, **(B)** the posterior arm of the internal capsule, and **(C)** the thalamo-mesencephalic junction.

In March 2018, a 59-year-old Caucasian man presented to the Emergency Department reporting an 8-month history of proximal leg weakness causing walking difficulties and decreased performances during bike races. He also experienced erratic muscular contractions assessed by his physiotherapist in October 2017. He complained about nocturnal cramps without any notion of muscular group or lateralization from the last 3 months. He was also dysarthric over the last month before hospital admission. After the first episode of speech assessment disturbance in February 2018, he underwent brain magnetic resonance imaging (MRI) and electroencephalogram (EEG), which were unremarkable. He first denied the use of any kind of stimulating drugs. There was also no history of dental paste application. He did not smoke and consumed alcohol occasionally. Clinical examination showed lower limb paresis with four limbs hyperreflexia, bilateral unsustained clonus, and dysarthria. Electromyography (EMG) showed chronic, moderate-to-severe, neurogenic changes with fasciculation potentials of four limbs and genioglossus muscles, compatible with a motor neuron disease. Motor-evoked potential (MEP) exploring the integrity of the descending motor pathways showed a discrepancy between a normal examination for the upper limbs and the abnormal cortico-spinal transmission in the lower limbs. In April 2018, after new symptoms of electric-like pain in his left foot, a clinical examination reported hypopallesthesia of the lower limbs not including iliac crests. The patient was questioned again about his lifestyle. He had no history of professional exposure to any specific toxin. He reported the consumption of vitamins and dietary supplements to improve his bike race performance. He took one portion of the dietary supplement daily from March 2018 to January 2019 (6.1 mg/100 g of zinc; 0.91 mg/100 g of copper). He had no history of erythema migrans nor excessive sweating. Somatosensory-evoked potential (SEP) showed the absence of cortical activities in the right hemisphere after the stimulation of the posterior tibial nerve. Spinal cord magnetic resonance imaging (MRI) showed abnormal linear hyperintensities on the T2-weighted views within the postero-lateral and posterior columns from C4 to T1 mimicking metabolic deficiency (Figure 1). Brain MRI showed bilaterally an abnormal mild hypersignal intensity of the cortico-spinal tract from the coronara radiata to the mesencephalic area (Figure 2) on the T2/FLAIR views (Figure 2). The diagnosis of degenerative or toxic myelopathy was suspected, and further investigations were performed. Routine laboratory workups including hematological (i.e., no signs of macrocytic or microcytic anemia), inflammatory, infectious, and auto-immune parameters were within normal range. Cobalamin, folate, vitamin E, and homocysteine levels were normal. Cerebrospinal fluid analysis was aspecific. Serum copper and ceruloplasmin levels were low [49 μg/dl (normal range: 70–140) and 0.16 g/L (0.20–0.60)], respectively, with normal serum zinc. Urinary copper excretion was 5.5 μg/24 h (< 35.0). Relative exchangeable copper [calculated as exchangeable (free) copper/total copper] was low at 16.1% (free copper 1.47 mmol/L).

### Copper absorption and supplementation

**Figure 3:**
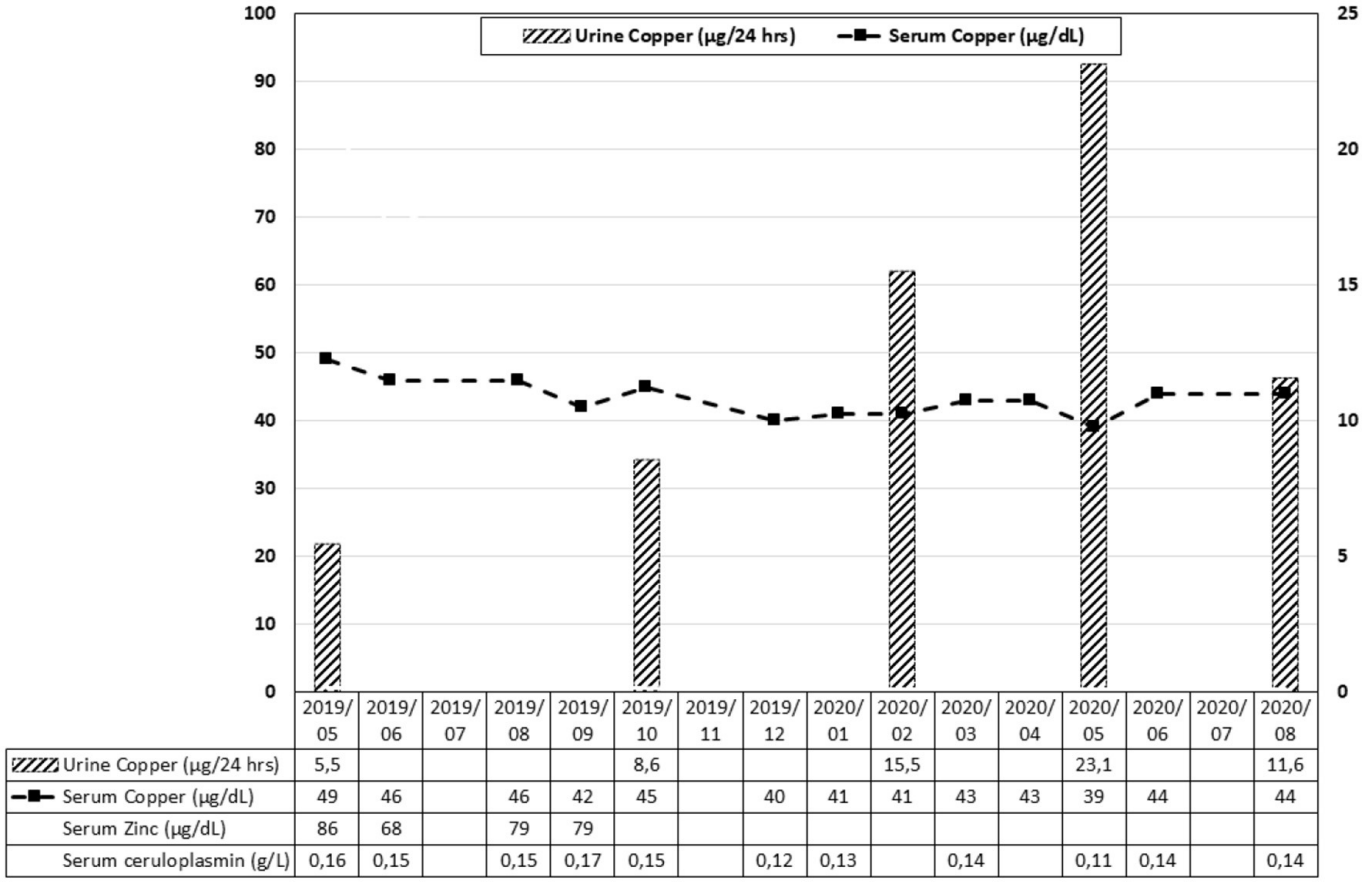
Evolution of serum copper, zinc, ceruloplasmin, and urine copper over time. The corresponding dosage regimen of copper supplementation is mentioned in the text.

Oral supplementation of copper was started after monitoring serum levels of copper and ceruloplasmin, and copper urinary excretion (Figure 3). The initial (May 2019) dosage regimen for copper supplementation was as follows: 8 mg copper sulfate pentahydrate daily for 1 week (corresponding to 2 mg of copper element), with a tapering of 2 mg every week to achieve a daily maintenance dose of 2 mg from the fourth week. In September 2019, as it appeared that copper and ceruloplasmin levels remained low, copper sulfate pentahydrate administration was increased to 16 mg daily. In November 2019, intravenous copper therapy was started using a hospital pharmacy preparation of copper sulfate corresponding to 2 mg elemental copper over eight consecutive days, thereafter 4 mg over the next 5 days, and ultimately followed by three infusions each week. In April 2020, the hospital pharmacy preparation was replaced by a commercial preparation of copper chloride dihydrate (0.4 mg copper element/ml). The patient received three infusions per week of the equivalent of 4 mg of the copper element. He was also maintained on 16 mg oral copper sulfate pentahydrate. Urinary copper excretion slightly increased during intravenous administration but fell again after discontinuation of the therapy in June 2020. A comprehensive gastro-intestinal workup (functional tests of intestinal absorption and intestinal biopsies) failed to reveal any sign of intestinal malabsorption. Next-generation whole-genome sequencing failed to reveal any known mutation of genes linked with copper homeostasis dysfunction, such as *ATP7A* (involved in *ATP7A*-distal motor neuropathy), *ATP7B* (thereby excluding a heterozygous status for Wilson's disease), or *SLC31A1* and *SLC31A2* genes encoding for copper transporters 1 and 2 (CTR1 and CTR2) in the intestinal mucosa, respectively. There were no *SOD1*-gene mutations, ruling out familial ALS, and the patient had a class III variant for *CP: c.1430C*>*T; p.(Pro477Leu)*.

### Clinical outcome

The patient's neurological status progressively worsened after June 2020, with gait disturbances and swallowing impairment. Clinically, amyotrophy of the thenar eminence was noted together with fasciculations in the left shoulder. The suspicion of amyotrophic lateral sclerosis (ALS) was strongly supported by EMG findings. Further evolution was dominated by a progression of symptoms resembling ALS, with spasticity and amyotrophy of the lower limbs, inability to walk, and dysarthria. Copper supplementation was interrupted in January 2021. In September 2021, blood or urine determinations of arsenic, cadmium, manganese, mercury, lead, and thallium were within normal range.

## Discussion

This case illustrates both the challenging diagnosis of copper-related neurological diseases and the difficulties of copper oral supplementation.

Indeed, the first clinical presentation of the patient, together with the results of the biological and radiological investigations, was consistent with a copper deficiency-related myelopathy (8–10). However, despite sustained copper supplementation, either orally or intravenously, serum copper and ceruloplasmin level never increased. The question arose whether this was reflecting a true copper deficiency at the cellular level, with some individuals in the general population remaining asymptomatic with low serum copper levels. Furthermore, the clinical course worsened to a more striking evidence of a motor neuron disease, with ALS as the most likely final diagnosis. The true impact of copper deficiency in triggering the initial myelopathy and subsequent progression toward ALS could be debated. In the few past years, copper deficiency has been demonstrated to result in various neurological conditions like isolated peripheral neuropathy, motor neuron disease, myelopathy, cerebral demyelination, cognitive impairment, and optic nerve neuropathy. Those symptoms can occur without any hematologic abnormalities. Spinal cord magnetic resonance imaging (MRI) often shows an extensive abnormal hypersignal intensity within the dorsal columns of the cord mimicking subacute combined degeneration (SCD) due to cobalamin deficiency from nitrous oxide toxicity (10, 11).

Inherited disorders of copper metabolism, like Menkes disease, Occipital Horn syndrome, X-Linked Distal Hereditary Motor Neuropathy, MEDNIK syndrome, Wilson disease, Aceruloplasminemia, Deficiency of the Copper Chaperone for superoxide dismutase, familial ALS, and deficiency of the cytochrome C oxidase assembly protein are known to induce a large panel of neurological manifestations (12) (specific genes and MRI patterns are detailed in Supplementary Table 1). Primary acquired copper deficiency is a rare condition due to its wide availability in aliments (4, 13). In our patient, as in other cases, the etiology of copper deficiency remained undetermined. The patient had ingested dietary supplements containing zinc for a couple of months, but low serum copper and ceruloplasmin level (together with low urinary excretion) persisted after discontinuation of zinc intake. As mentioned earlier, the most frequent cause of copper deficiency is intestinal malabsorption (2–4). Common causes of intestinal malabsorption were reasonably excluded by functional tests and biopsies of the small bowel. There are no definitive guidelines regarding the dose, duration, route, and form of copper supplementation. Experts recommend 8 mg of elemental copper each day orally for a week, 6 mg for the second week, 4 mg for the third week, and 2 mg thereafter (4). Parenteral administration is the preferred route in the presence of intestinal malabsorption. In our patient, the oral route failed to achieve any increase in serum copper level, and intravenous administration only resulted in a modest increase in urinary copper excretion. Genetic causes of copper intestinal malabsorption were extensively investigated and remained negative. Regarding biological monitoring, serum copper levels are expected to decrease early after the initial peak following intravenous copper administration, as copper is rapidly stored in the liver before it bound to ceruloplasmin in the plasma. Also, urinary copper excretion mainly reflects that copper is not binding to ceruloplasmin, therefore potentially in excess or even toxic.

Investigations on the relationship between the development of ALS and trace metal levels, including copper and zinc, have led to conflicting results, but with a trend for lower serum copper and ceruloplasmin levels in patients with ALS compared to a control group (14–19).

It remains to be discussed if the patient presented two different diseases, copper deficiency-related myelopathy and ALS, or if this was a single entity with motor neuron disease as a late complication of copper deficiency. In 2006, Weihl et al. reported three cases of patients with copper deficiency who had clinical and electrodiagnostic evidence of lower motor neuron disease mimicking ALS (20). Although the clinical criteria are met and imply the presence of an ALS-type motor neuron pathology, the commonly used diagnostic criteria do not appear to be completely met, given the probable coexistence of two pathologies linked to motor neuron impairment (21). It is suggested that SOD1-linked ALS would be related to misfolding or/and aggregation of this enzyme, which may be caused due to the inability of the protein to bind with copper, preventing its antioxidant faculties (22), while it is already known that the copper and ceruloplasmin levels do not change as expected in healthy population in non-genetic ALS (14–19). Apart from ALS, copper dyshomeostasis has been implicated in other neurodegenerative disorders' pathogenesis (23). In other types of motor neuron impairment, those inabilities could be due to copper homeostasis disorder, in terms of absorption, transport, or various enzymatic activity impairment, such as SOD1 (24). The genetic polymorphism found in our patient in *CP*, coding for ceruloplasmin, might have been a facilitating factor leading to the clinical development of the copper deficiency-related neurological disorder in association with zinc supplements (25).

## Conclusion

This case illustrates the trouble of meeting ALS diagnostic criteria, while another motor neuron impairment-linked pathology is associated. The diagnosis of copper deficiency-related neurological disorders remains challenging. As asymptomatic patients may present with relatively low serum copper levels, the assessment of true pathogenicity of the copper deficiency remains questionable, in particular, in patients presenting symptoms of copper deficiency-related myelopathy and/or ALS. Reasons for the persistence of low serum copper levels despite heavy supplementation cannot always be identified, and copper-related genetic polymorphism might be investigated.

## Data availability statement

The raw data supporting the conclusions of this article will be made available by the authors, without undue reservation.

## Author contributions

AB and AI: patient follow-up and writing. TW: review and comments. PB and VH: review. EW: genetic consulting. TD: imagery and review. PH: project manager and writing. All authors contributed to the article and approved the submitted version.
