# Supplementary material for: Case report: Motor neuron disease phenotype associated with symptomatic copper deficiency: Challenging diagnosis and treatment
Source: Front Neurol. 2023 Jan 4;13:1063803. doi: 10.3389/fneur.2022.1063803 (PMC9845570; doi:10.3389/fneur.2022.1063803)
Supplement: Supplementary file 1 [file Data_Sheet_1.PDF]

Supplement Table 1 : Clinical and paraclinical features of copper related neurological disorders

**Copper related neurological disorders**	***Gene*/protein implicated**	**Clinical features**	**Radiological findings**
Aceruloplasminemia[1,2]	Ceruloplasmin[3]	Dystonia/abnormal gait, dysarthria, dementia[3]	T2* high signal due to progressive iron accumulation involving the retina and basal ganglia[3]
Amyotrophic lateral sclerosis (familial form)[1]	SOD1[4]	Adult-onset progressive muscle weakness[4]	MRI : T2 pyramidal tracts hyperintensity[4], T1 tongue hyperintensity in bulbar form[5]
Deficiency of the Cytochrome C Oxidase Assembly Protein SCO1[1]	Heterozygosity for *SCO1* gene mutations[6]	Neonatal-onset hepatic failure and ketoacidotic comas[6]	No specific MRI pattern
Deficiency of the Cytochrome C Oxidase Assembly Protein SCO2[1]	*SCO2*[7]	Hypertrophic cardio-myopathy and encephalopathy[7]	+/- Brainstem or cerebellar abnormalities seen on MRI[7,8]
Menkes disease[1]	*ATP7A*[9,10]	Feeding difficulties, hypotonia progressing to hypertonia and	MRI : cerebral atrophy, ventriculomegaly, and cerebellar atrophy and

		seizures (including infantile spasms with hypsarrhythmia)[9]	focal lesions in the basal ganglia[10]
MEDNIK syndrom[1]	*AP1S1*[11,12]	Mental retardation, Enteropathy, Deafness, peripheral Neuropathy, Ichthyosis, and Keratoderme[11,12]	MRI : brain atrophy, T2 hyperintensity of the basal ganglia, especially caudate and putamen[11,12]
Occipital horn syndrome[1]	*ATP7A*[13]	Mild allelic variant of Menkes[13]	No specific MRI pattern
Wilson disease[1,2]	*ATP7B*[14]	Movement and behavior/personality disorders[14]	MRI T2 images of high signal intensity in the tegmentum except for the red nucleus, with preservation of the signal intensity of the lateral portion of the pars reticulata of the substantia nigra and hypointensity of the superior colliculus (Panda Sign). Cerebral atrophy[15]
X-Linked Distal Hereditary Motor Neuropathy[1]	*ATP7A*[13]	Distal muscular atrophy and weakness in older children or adults[13]	No specific MRI pattern

Abbreviations :

MRI – Magnetic resonance imaging ; SOD1 – Super Oxyde Dismutase 1 ; SCO1,2 - Synthesis Of Cytochrome C Oxidase 1,2; *ATP7A/B* – ATPase copper-transporting ATPase ½ ; MEDNIK - Mental retardation, Enteropathy, Deafness, peripheral Neuropathy,Ichthyosis, and Keratoderma ; *AP1S1*- Adaptor Related Protein Complex 1 Subunit Sigma 1 ;

References :

1) Desai V, Kaler SG. Role of copper in human neurological disorders. Am J Clin Nutr. 2008 Sep;88(3):855S-8S. doi: 10.1093/ajcn/88.3.855S

2) Clayton P, Tuschl K. Metal Storage Disorders: Inherited Disorders of Copper and Manganese Metabolism and Movement Disorders. In: Ebrahimi-Fakhari, D and Pearl, P, (eds.) Movement Disorders and Inherited Metabolic Disorders: Recognition, Understanding, Improving Outcomes. Cambridge University Press: Cambridge, UK. 2020. 230-43.

3) Miyajima H, Hosoi Y. Aceruloplasminemia. GeneReviews®. 2003;Aug 12 (updated Sep 27, 2018)

4) Simon NG, Turner MR, Vucic S et-al. Quantifying disease progression in amyotrophic lateral sclerosis. Ann. Neurol. 2014;76 (5): 643-57. doi:10.1002/ana.24273

5) Fox M & Cohen A. "Bright Tongue Sign" in ALS. Neurology. 2012;79(14):1520. doi:10.1212/wnl.0b013e31826d5ffc

6) Valnot I, Osmond S, Gigarel N, et al. Mutations of the SCO1 gene in mitochondrial cytochrome c oxidase deficiency with neonatal-onset hepatic failure and encephalopathy. Am J Hum Genet. 2000;67(5):1104–9.

7) Jaksch M, Ogilvie I, Yao J, et al. Mutations in SCO2 are associated with a distinct form of hypertrophic cardiomyopathy and cytochrome c oxidase deficiency. Hum Mol Genet. 2000;9(5):795–801.

8) Pronicki M, Kowalski P, Piekutowska-Abramczuk D, Taybert J, Karkucinska-Wieckowska A, Szymanska-Debinska T, Karczmarewicz E, Pajdowska M, Migdal M, Milewska-Bobula B, Sykut-Cegielska J, Popowska E. A homozygous mutation in the SCO2 gene causes a spinal muscular atrophy like presentation with stridor and respiratory insufficiency. Eur J Paediatr Neurol. 2010 May;14(3):253-60. doi: 10.1016/j.ejpn.2009.09.008. Epub 2009 Oct 29. PMID: 19879173.

9) Cao B, Yang X, Chen Y, et al. Identification of novel ATP7A mutations and prenatal diagnosis in Chinese patients with Menkes disease. Metab Brain Dis. 2017;32(4):1123–31.

10) Manara R, Rocco MC, D'Agata L, et al. Neuroimaging changes in Menkes disease: Part 2. AJNR Am J Neuroradiol. 2017;38(10):1858–65

11) Martinelli D, Travaglini L, Drouin CA, et al. MEDNIK syndrome: A novel defect of copper metabolism treatable by zinc acetate therapy. Brain. 2013;136(Pt 3):872–81. 13.

12) Martinelli D, Dionisi-Vici C. AP1S1 defect causing MEDNIK syndrome: A new adaptinopathy associated with defective copper metabolism. Ann N Y Acad Sci. 2014;1314:55–63.

13) van Hasselt PM, Clayton PT, Houwen RHJ. Disorders of the transport of copper, iron, magnesium, manganese, selenium and zinc. In Saudubray J-M, Baumgartner MR, Walter J, editors. Inborn Metabolic Diseases. Berlin: Springer; 2016, pp. 531–50.

14) Gow PJ, Smallwood RA, Angus PW, Smith AL, Wall AJ, Sewell RB. Diagnosis of Wilson's disease: an experience over three decades. Gut. 2000 Mar;46(3):415-9. doi: 10.1136/gut.46.3.415. PMID: 10673307; PMCID: PMC1727845.

15) Singh P, Ahluwalia A, Saggar K, Grewal CS. Wilson's disease: MRI features. J Pediatr Neurosci. 2011 Jan;6(1):27-8. doi: 10.4103/1817-1745.84402. PMID: 21977083; PMCID: PMC3173909.
